# Podiatrists’ perceptions of diabetic foot care services in a Scottish NHS health board area

**DOI:** 10.1186/1757-1146-8-S1-A12

**Published:** 2015-04-20

**Authors:** Jennifer Scott, Gordon Hendry, Debbie Turner, Stuart Baird, Ruth Barn

**Affiliations:** 1 School of Health and Life Sciences, Glasgow Caledonian University, UK

## Aims/Objectives

People with diabetes are at a significantly increased risk of foot ulceration, infection, and lower extremity amputation. The Scottish Intercollegiate Guidelines Network1 (SIGN) published guidelines on the care and management of foot complications in patients with diabetes in 2010. Little data is available on the extent to which these guidelines have been implemented, or on the perceptions of podiatrists working to the guidelines in this sector. The aims of this study were to 1) elicit podiatrists’ perceptions of the diabetic foot care service provided in the health board area, 2) identify whether or not podiatrists perceived that national diabetic foot care guidelines are being met, and 3) identify any perceived barriers to optimal diabetic foot care.

## Content

A mixed (quantitative and qualitative) methods approach was adopted. An anonymous cross-sectional survey of diabetic foot care service provision was administered to podiatrists attending a professional development event. Survey questions were formulated to address key areas of importance outlined in national diabetic foot care guidelines. Deeper exploration of podiatrists’ perceptions of the provision of diabetic foot care services was conducted through a focus group using an interpretative phenomenological approach with thematic analysis. Survey data was summarised using descriptive statistics to identify areas of adherence to, or deviation from recommended clinical practice.

## Outcomes

Fifty-nine participants who currently manage diabetic patients as part of their caseload took part in the survey (response rate 40%), and nine participated in the focus group.

## Relevance

This research highlights several areas for improvement in the delivery of diabetic foot care services across the NHS health board under study, as well as some examples of good, effective practice. It is likely that the findings will be of interest to service managers across Scotland and the wider podiatric community as they seek to deliver optimal patient care with limited resources.

## Discussion

The survey suggested that clinical practice adhered to certain guideline recommendations in the recording of patient risk electronically, appropriate referrals to multi-disciplinary teams and offloading of ulcers. It indicated that time constraints were the most commonly identified barrier to complying with the official care guidelines. Less than half of respondents (42%) believed that all SIGN guidelines were being met, and less than half (44%) stated they believed screening and assessment targets were being met. Analysis of the qualitative data revealed inadequacies in current risk stratification procedures, barriers to accessing certain services and challenges achieving effective patient education.

## Conclusions

NHS publications indicate screening targets are being met, however the research indicates that podiatrists do not perceive this to be the case. It is unclear whether this perception is justified. A cross-section of podiatrists in the focus group indicated that screening may not be the most appropriate use of resources.

Further research is needed to identify the most effective solutions to the issues raised.
